# Editorial: Microbiology of Ethnic Fermented Foods and Alcoholic Beverages of the World

**DOI:** 10.3389/fmicb.2017.01377

**Published:** 2017-07-20

**Authors:** Jyoti P. Tamang, Wilhelm H. Holzapfel, Dong H. Shin, Giovanna E. Felis

**Affiliations:** ^1^Microbiology, Sikkim University Gangtok, India; ^2^Microbiology, Handong Global University Pohang, South Korea; ^3^Microbiology, Chonbuk National University Jeonju, South Korea; ^4^Microbiology, University of Verona Verona, Italy

**Keywords:** editorial, fermented foods, food microbiology, next generation sequence, global

Approximately there may be around 5,000 varieties of common and uncommon fermented foods and alcoholic beverages in the world. Global fermented foods are classified into 9 major groups on the basis of substrates (raw materials) used from plant/animal sources: fermented cereals, fermented vegetables and bamboo shoots, fermented legumes, fermented roots/tubers, fermented milk products, fermented and preserved meat products, fermented, dried, and smoked fish products, miscellaneous fermented products, and alcoholic beverages. Fermented foods are the hubs of consortia of microorganisms, which transform the chemical constituents of raw materials of plant/animal sources during *in situ*/*ex situ* fermentation, thereby enhance the nutritional value with health-promoting bioactive compounds to consumers.

Common genera of the lactic acid bacteria isolated from various fermented foods globally are *Alkalibacterium, Carnobacterium, Enterococcus, Lactobacillus, Lactococcus, Leuconostoc, Oenococcus, Pediococcus, Streptococcus, Tetragenococcus, Vagococcus* and *Weissella*. Species of Bacillus are reported for alkaline-fermented foods of Asia and Africa. The association of several species of *Kocuria, Micrococcus* (members of the Actinobacteria), and *Staphylococcus* (belonging to the Firmicutes) has been reported for fermented milk, fermented meat, and fish products. Species of *Bifidobacterium, Brachybacterium, Brevibacterium*, and *Propionibacterium* have been isolated from cheese and species of *Arthrobacter* and *Hafnia* from meat fermentation. *Enterobacter cloacae, Klebsiella pneumoniae, K. pneumoniae* subsp. *ozaenae, Haloanaerobium, Halobacterium, Halococcus, Propionibacterium*, and *Pseudomonas*, are also present in numerous fermented foods.

Genera of yeasts reported for fermented foods, alcoholic beverages, and non-food mixed amylolytic starters are *Brettanomyces, Candida, Cryptococcus, Debaryomyces, Dekkera, Galactomyces, Geotrichum, Hansenula, Hanseniaspora, Hyphopichia, Issatchenkia, Kazachstania, Kluyveromyces, Metschnikowia, Pichia, Rhodotorula, Rhodosporidium, Saccharomyces, Saccharomycodes, Saccharomycopsis, Schizosaccharomyces, Sporobolomyces, Torulaspora, Torulopsis, Trichosporon, Yarrowia*, and *Zygosaccharomyces*. Major roles of filamentous fungi in fermented foods and alcoholic beverages are mainly production of enzymes and also degradation of anti-nutritive factors. Species of *Actinomucor, Amylomyces, Aspergillus, Monascus, Mucor, Neurospora, Parcilomyces, Penicillium, Rhizopus*, and *Ustilago* are reported for many fermented foods, Asian non-food amylolytic starters and alcoholic beverages.

Direct DNA extraction from samples of fermented foods, commonly called culture-independent methods, is nowadays frequently used in food microbiology to profile both cultivable and uncultivable microbial populations from fermented foods. Amplified ribosomal DNA restriction analysis (ARDRA) and denaturing gradient gel electrophoresis (DGGE) techniques developed to profile microbial communities directly from fermented foods, and are based on sequence-specific distinctions of 16S rDNA or 26S rDNA amplicons produced by PCR.

Application of next generation sequencing (NGS) such as metagenomic approaches by using parallel pyrosequencing of tagged 16S rRNA gene amplicons provide information on microbial communities as profiled in kimchi, a naturally fermented vegetable product of Korea, nukadoko, a fermented rice bran of Japan, narezushi, a fermented salted fish and cooked rice of Japan, and ben-saalga, a traditional gruel of pearl millet of Burkina Faso. A proteomics identification method based on protein profiling using matrix-assisted laser desorption ionizing-time of flight mass spectrometry (MALDI-TOF MS) is used to identify bacteria in fermented foods. NGS has revealed the new dimension of microbial ecology comprising both cultivable and uncultivable microorganisms in many ethnic fermented foods and beverages of the world.

This e-book is a compilation of 15 originals and reviews papers written by 94 authors. We tried to represent the main Asian, African, European, and American fermented foods and alcoholic beverages.

## Author contributions

JT prepared a draft concept on Resource Topic of the present e-book and list of authors for papers, supplemented and corrected by WH, DS, and GF. JT was the main corresponding Editor, however, WH, DS, and GF also helped to provide the names of referrers, etc.

### Conflict of interest statement

The authors declare that the research was conducted in the absence of any commercial or financial relationships that could be construed as a potential conflict of interest.

